# Outcome Measures in Rheumatology - Interventions for medication Adherence (OMERACT-Adherence) Core Domain Set for Trials of Interventions for Medication Adherence in Rheumatology: 5 Phase Study Protocol

**DOI:** 10.1186/s13063-018-2565-z

**Published:** 2018-03-27

**Authors:** Ayano Kelly, Allison Tong, Kathleen Tymms, Lyn March, Jonathan C. Craig, Mary De Vera, Vicki Evans, Geraldine Hassett, Karine Toupin-April, Bart van den Bemt, Armando Teixeira-Pinto, Rieke Alten, Susan J. Bartlett, Willemina Campbell, Therese Dawson, Michael Gill, Renske Hebing, Alexa Meara, Robby Nieuwlaat, Yomei Shaw, Jasvinder A. Singh, Maria Suarez-Almazor, Daniel Sumpton, Peter Wong, Robin Christensen, Dorcas Beaton, Maarten de Wit, Peter Tugwell, On behalf of the OMERACT-Adherence Group, Marieke Scholte-Voshaar, Khoula AlMaqbali, Annica Barcenilla-Wong, Peter Cheung, Luke Crimston-Smith, Marita Cross, Rebecca Davey, Paul Emery, Kieran Fallon, Sarah Flint, David Graham, Stephen Hall, Susan Hermann, Helen Keen, Katerina Koutsogianni, Irwin Lim, Francois Nantel, Sean O’Neill, Clare O’Sullivan, Premarani Sinnathurai, Biljana Zeljkovic, Ayano Kelly, Allison Tong, Kathleen Tymms, Lyn March, Mary De Vera, Vicki Evans, Geraldine Hassett, Karine Toupin-April, Bart van den Bemt, Marieke Scholte-Voshaar, Susan Bartlett, Peter Tugwell, Lyn March, Rieke Alten, Willemina Campbell, Therese Dawson, Michael Gill, Renske Hebing, Alexa Meara, Robby Nieuwlaat, Yomei Shaw, Jasvinder A. Singh, Maria Suarez-Almazor, Daniel Sumpton, Peter Wong, Robin Christensen, Dorcas Beaton, Maarten de Wit

**Affiliations:** 1 Canberra Rheumatology, Level 9, 40 Marcus Clarke St, Canberra City, ACT 2606 Australia; 2 Department of Rheumatology, Canberra Hospital, Canberra, ACT Australia; 3 College of Health and Medicine, Australian National University, Canberra, ACT Australia; 4 Centre for Kidney Research, The Children’s Hospital at Westmead, Sydney, NSW Australia; 5 Sydney School of Public Health, The University of Sydney, Sydney, NSW Australia; 6 Department of Rheumatology, Royal North Shore Hospital, Sydney, NSW Australia; 7 Institute of Bone and Joint Research, Kolling Institute of Medical Research, Sydney, NSW Australia; 8 Northern Clinical School, The University of Sydney, Sydney, NSW Australia; 9 Faculty of Pharmaceutical Sciences, The University of British Columbia, Vancouver, BC Canada; 10 Arthritis Research Centre of Canada, Richmond, BC Canada; 11 Patient Research Partner, Clear Vision Consulting, Canberra, ACT Australia; 12 Department of Rheumatology, Liverpool Hospital, Sydney, NSW Australia; 13 Ingham Institute of Applied Medical Research, Sydney, NSW Australia; 14 Children’s Hospital of Eastern Ontario Research Institute, Ottawa, ON Canada; 15 Department of Pediatrics and School of Rehabilitation Sciences, University of Ottawa, Ottawa, ON Canada; 16 Department of Pharmacy, Sint Maartenskliniek, Ubbergen, Netherlands; 17 Radboud University Medical Centre, Nijmegen, Netherlands; 18 Department of Rheumatology, Clinical Immunology, Osteology, Physical therapy and Sports Medicine, Schlosspark Klinik, Charité University Medicine, Berlin, Germany; 19 Department of Medicine, McGill University, Montreal, Canada; 20 Division of Rheumatology, Johns Hopkins School of Medicine, Baltimore, MD USA; 21 Patient Research Partner, Toronto Western Hospital, Toronto, Ottawa Canada; 22 Lord Street Specialist Centre, Port Macquarie, NSW Australia; 23 Mayo Hospital Specialist Centre, Taree, NSW Australia; 24 Patient Research Partner, Dragon Claw, Sydney, NSW Australia; 25 Amsterdam Rheumatology and Immunology Centre, Amsterdam, Netherlands; 26 The Ohio State University, Wexner Medical Center, Columbus, OH USA; 27 Department of Clinical Epidemiology and Biostatistics, McMaster University, Hamilton, ON Canada; 28 National Data Bank for Rheumatic Diseases, Wichita, KS USA; 29 Medicine Service, VA Medical Center, Birmingham, AL USA; 30 Department of Medicine, School of Medicine, University of Alabama, Birmingham, AL USA; 31 Division of Epidemiology, School of Public Health, University of Alabama, Birmingham, AL USA; 32 Section of Rheumatology and Clinical Immunology, Department of General Internal Medicine, The University of Texas MD Anderson Cancer Center, Houston, TX USA; 33 Department of Rheumatology, Concord Hospital, Sydney, NSW Australia; 34 Mid-North Coast Arthritis Clinic, Coffs Harbour, NSW Australia; 35 University of New South Wales Rural Clinical School, Coffs Harbour, NSW Australia; 36 Musculoskeletal Statistics Unit, The Parker Institute, Bispebjerg and Frederiksberg Hospital, Copenhagen, Denmark; 37 Musculoskeletal Health & Outcomes Research, Li Ka Shing Knowledge Institute, St. Michael’s Hospital, Toronto, ON Canada; 38 Institute for Work & Health, Toronto, ON Canada; 39 Department of Occupational Science & Occupational Therapy and the Institute of Health Policy Management & Evaluation, University of Toronto, Toronto, ON Canada; 40 Metamedica, VU Medical Centre, Amsterdam, The Netherlands; 41 Department of Medicine, University of Ottawa, Ottawa, ON Canada

**Keywords:** Core domain set, Outcomes research, Patient-centred outcomes, Clinical trials, Rheumatology, Medication Adherence, Adherence, Compliance, Persistence

## Abstract

**Background:**

Over the last 20 years, there have been marked improvements in the availability of effective medications for rheumatic conditions such as gout, osteoporosis and rheumatoid arthritis (RA), which have led to a reduction in disease flares and the risk of re-fracture in osteoporosis, and the slowing of disease progression in RA. However, medication adherence remains suboptimal, as treatment regimens can be complex and difficult to continue long term. Many trials have been conducted to improve adherence to medication. Core domains, which are the outcomes of most relevance to patients and clinicians, are a pivotal component of any trial. These core domains should be measured consistently, so that all relevant trials can be combined in systematic reviews and meta-analyses to reach conclusions that are more valid. Failure to do this severely limits the potential for trial-based evidence to inform decisions on how to support medication adherence. The Outcome Measures in Rheumatology (OMERACT) – Interventions for Medication Adherence study by the OMERACT-Adherence Group aims to develop a core domain set for interventions that aim to support medication adherence in rheumatology.

**Methods/design:**

This OMERACT-Adherence study has five phases: (1) a systematic review to identify outcome domains that have been reported in interventions focused on supporting medication adherence in rheumatology; (2) semi-structured stakeholder interviews with patients and caregivers to determine their views on the core domains; (3) focus groups using the nominal group technique with patients and caregivers to identify and rank domains that are relevant to them, including the reasons for their choices; (4) an international three-round modified Delphi survey involving patients with diverse rheumatic conditions, caregivers, health professionals, researchers and other stakeholders to develop a preliminary core domain set; and (5) a stakeholder workshop with OMERACT members to review, vote on and reach a consensus on the core domain set for interventions to support medication adherence in rheumatology.

**Discussion:**

Establishing a core domain set to be reported in all intervention studies undertaken to support patients with medication adherence will enhance the relevance and the impact of these results and improve the lives of people with rheumatic conditions.

**Electronic supplementary material:**

The online version of this article (10.1186/s13063-018-2565-z) contains supplementary material, which is available to authorized users.

## Background

Musculoskeletal conditions are a major cause of disability worldwide and a burden on individuals and health-care systems [1]. Advances in drug development throughout the 21st century have led to a dramatic improvement in outcomes for patients with rheumatic conditions [2, 3]. Conditions such as gout, osteoporosis and rheumatoid arthritis (RA) are amongst the most common rheumatic conditions that require long-term use of medications to improve morbidity, mortality and other health outcomes [4–7]. However, rates of medication adherence have been reported to be as low as 10% in gout, 30% in RA and 45% in osteoporosis [8–10]. Barriers to medication adherence include perceptual barriers (e.g. concerns about side effects and uncertainty regarding the efficacy of medications) and practical barriers (e.g. forgetfulness, inconvenience and cost) [11–14].

Researchers most commonly support the use of the word ‘adherence’ in preference to ‘compliance’ or ‘concordance’ [15, 16]. ‘Adherence’ highlights the outcomes of a shared decision-making approach where the patient and physician agree upon a treatment plan that the patient will follow [17]. ‘Compliance’ may portray a negative paternalistic relationship between the health-care provider and the patient [15]. ‘Concordance’ emphasises a balanced therapeutic alliance between the patient and the health-care provider [18]; however, even when ‘concordance’ is successful, patients may alter or decide not to take their medicine [18]. Thus, ‘adherence’ remains the preferred term. While non-pharmacological management is an important aspect of many rheumatic conditions, adherence to non-pharmacological management is currently beyond the scope of this study.

**Fig. 1:**
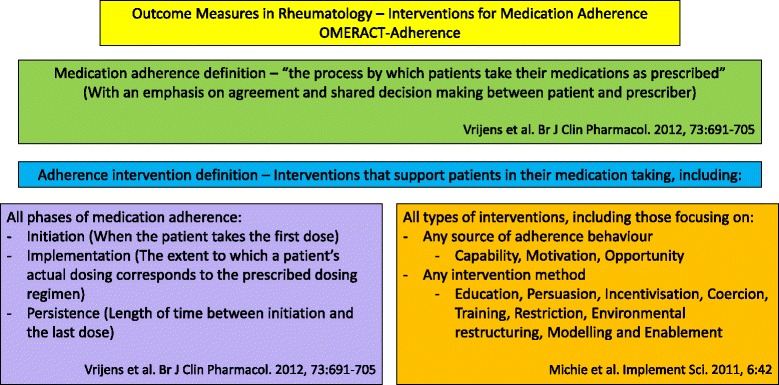
Scope and definitions of OMERACT-Adherence study

The ABC taxonomy of adherence [15, 19] defines adherence as ‘the process by which patients take their medications as prescribed’ and comprises: (a) initiation (when the patient takes the first dose of a prescribed medication), (b) implementation (the extent to which a patient’s actual dosing corresponds to the prescribed dosing regimen, from initiation until the last dose) and (c) persistence (the length of time between initiation and the last dose, which immediately precedes discontinuation, *i.e.,* when the patient stops taking the prescribed medication) [15]. The behaviour change wheel will be used to categorise intervention approaches relevant to improving adherence behaviours (Appendix 1) [19]. In the OMERACT-Adherence study, interventions may focus on any adherence phase (initiation, implementation or persistence), source of medication, adherence behaviour (capability, opportunity or motivation) and method (education, persuasion, incentivisation, coercion, training, restriction, environmental restructuring, modelling and enablement) (Fig. 1).

Adherence research plays an important role in bridging the chasm between recommended and best practice approaches to disease management to improve medication adherence. Clinical trials have been conducted in people with rheumatic conditions to resolve ambivalence and improve medication acceptance and adherence, and thereby enhance health outcomes [20]. Yet few interventions have demonstrated meaningful improvements in either medication adherence or clinical outcomes across medical specialties [20, 21]. A limitation in collating the results of these trials to identify successful interventions better is the lack of clarity of core outcomes and the wide variability in adherence measures. There is need for a consensus-based core domain set for interventions to improve medication adherence.

Worldwide, there have been many initiatives to develop core domain sets [22, 23], defined as the minimum set of outcome domains that should be measured and reported in clinical trials for a specific condition. The Outcome Measures in Rheumatology (OMERACT) initiative commenced in 1992 and has expanded to develop core domain sets in multiple rheumatic conditions [24]. There are now over 20 groups developing core domain sets for specific conditions [22, 25] and there are several methodological groups examining the core domains of interventions and measurements of outcomes that are relevant across rheumatic conditions, including health literacy, shared decision-making and work productivity [26–28].

**Fig. 2:**
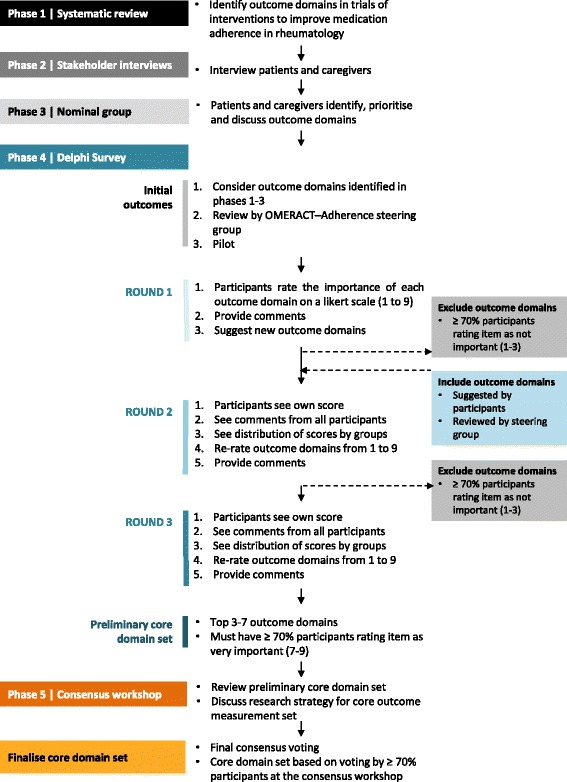
Conceptual schema of OMERACT-Adherence core domain set. ACR American College of Rheumatology

The OMERACT-Adherence Group aims to establish a core domain set for clinical trials to support medication adherence in patients with rheumatic conditions of all ages (Fig. 2). The OMERACT-Adherence Group was established in December 2016, and comprises over 40 members from 11 countries: Australia, Canada, Germany, Greece, the Netherlands, Singapore, the United Kingdom, Oman, Switzerland, Denmark and the United States. The members include patients, rheumatologists, nurses, pharmacists, behavioural scientists, occupational therapists, industry representatives, researchers in outcomes and medication adherence, and clinical trialists. The patient perspective is highly valued and integrated into all OMERACT activities, as the ultimate aim is to improve clinical outcomes for patients [29]. Patient research partners are members of the steering committee of the OMERACT-Adherence Group and help with the design, conduct, analysis and dissemination of all studies.

The five specific objectives of this OMERACT-Adherence study are to: (1) conduct a systematic literature review to describe the scope and consistency of domains used in rheumatology interventions addressing medication adherence; (2) identify additional domains that are important to patients and their caregivers and elucidate the reasons for their choices; (3) ascertain the perspectives of other stakeholders including health professionals, researchers, purchasers, payers, policymakers and industry representatives on core domains; (4) develop a preliminary core domain set for clinical trials with input from all stakeholder groups and (5) seek a consensus on the OMERACT-Adherence core domain set by a ballot of the OMERACT members.

## Methods/design

**Fig. 3:**
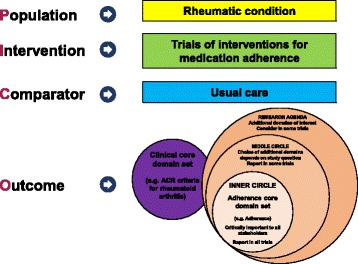
OMERACT-Adherence study process

The OMERACT-Adherence study methodology is adapted from the OMERACT framework, which is recognised as a valid approach for establishing a core domain set [22]. The protocol includes a SPIRIT checklist for recommended items to address in a clinical trial protocol and related documents (Additional file 1). The proposed scope of work to achieve the five OMERACT-Adherence study objectives is outlined in Fig. 3.

### Phase 1: systematic review of outcome domains and measures reported in trials of medication adherence

A systematic review will be conducted to identify and compare outcome domains and measures reported in interventions to improve medication adherence in rheumatology clinical trials. Outcome domains are the name of the broad concept that is measured (e.g. adherence, medication knowledge and medication skill). An outcome is the specific result in a domain arising from exposure to a causal factor or a health intervention (e.g. disease-modifying anti-rheumatic drug knowledge in RA and self-injection skill). An outcome measure includes the specific measurement instrument (the tool to measure a quality or quantity of a variable, e.g. pill count), the specific metric (e.g. a change from baseline) and the method of aggregation (e.g. mean or median for continuous measures or proportion for categorical measures) [30, 31].

#### Search strategy

Electronic databases (MEDLINE, Embase, PsycINFO, CINAHL and CENTRAL) will be searched to 31 October 2017 to identify all trials of interventions aiming to improve medication adherence involving patients with rheumatic conditions. The search will use medical subject headings for concepts including ‘patient compliance’, ‘medication adherence’, ‘intervention’, ‘inflammatory arthritis’, ‘rheumatoid arthritis’, ‘psoriatic arthritis’, ‘ankylosing spondylitis’, ‘juvenile idiopathic arthritis’, ‘connective tissue diseases’, ‘systemic lupus erythematosus’, ‘vasculitis’, ‘Sjogren’s syndrome’, ‘osteoporosis’ and ‘gout’ and keywords for concepts that do not match. The bibliographies of included articles will be searched by hand.

#### Types of studies and interventions

All publications studying interventions aiming to improve medication adherence in rheumatic conditions will be included. Given the limited number of randomised controlled trials (RCTs) for medication adherence in rheumatic conditions [20], non-controlled and single-arm interventions for medication adherence in rheumatic conditions will be included.

#### Types of participants

Studies involving participants of all ages with any rheumatic condition, including inflammatory arthritis, connective tissue diseases and osteoporosis, will be included.

#### Exclusion criteria

Conference reports and abstracts will be excluded given their space constraints. For feasibility, the search will be restricted to English language articles.

#### Eligibility of studies

Two reviewers will independently screen the abstracts and full text of all potentially relevant studies. Any uncertainties on the eligibility of studies to be included will be resolved through a third reviewer.

#### Data extraction

Data will be extracted and entered into Microsoft Excel using a pre-designed form, piloted before full data extraction with a sample of included studies. The primary reviewer will extract the following from all included interventions: first author, date of publication, countries in which the trial was conducted, sample size, participant characteristics (age, gender, condition and medication) and trial duration. In addition, the type of intervention and all adherence-related outcomes reported in the trial will be extracted. Adherence-related outcomes include adherence, and any other outcomes related to adherence behaviour (including capability, opportunity and motivation) [19]. For each outcome, the definitions, outcome measures used, time points, metric and method of aggregation will be extracted. Clinical outcomes for specific conditions will not be extracted, as this work is already being undertaken by other OMERACT groups [24]. Clinical outcomes are defined as any outcome that would fall under the four core areas in the OMERACT filter of death, life impact, resource use and pathophysiological manifestations for the specific condition, and they also include adverse events [24].

#### Data analysis and presentation

Two reviewers will group similar outcomes into outcome domains, which will be reviewed and modified by the OMERACT-Adherence steering committee. The frequency of each domain and outcome measure reported across trials will be calculated. Domains and measures will be compared with those identified in the 2014 Cochrane Systematic Review of RCTs to enhance medication adherence, which includes 182 RCTs across other specialties [32].

### Phase 2: stakeholder interviews

Semi-structured interviews will be conducted with patients and caregivers to ascertain individual perspectives on outcome domains. The interview guide will incorporate findings from phase 1 and help to give a greater understanding of the values and beliefs that underlie candidate domains. Additional outcome domains will also be identified in this phase. We will follow the consolidated criteria for reporting qualitative research (COREQ) to guide our methods and reporting [33].

#### Participants and recruitment

Adults with gout, osteoporosis or RA and their caregivers (defined by the patient as a significant person or family member who is aware of the patient’s illness and treatments) will be eligible to participate in an interview. Three conditions have been chosen for the phase 2 interviews and phase 3 focus groups, based on feasibility. They represent common rheumatic conditions with known poor levels of adherence [8–10]. Patients with diverse rheumatic conditions will be included in phases 1, 4 and 5 to ensure the core domain set is applicable to all rheumatic conditions. Participants will be identified by treating rheumatologists at participating centres in Australia: Liverpool Hospital (NSW), Canberra Rheumatology (ACT and NSW), BJC Health (NSW) and Royal North Shore Hospital (NSW). Although this phase includes participants from one country only, all other phases will include participants from different countries. A purposive sampling technique will be applied to include a broad range of demographic characteristics (age, gender, socioeconomic status, educational level and ethnicity) and clinical characteristics (type, duration and severity of condition).

Based on our experience with previous qualitative interview studies, target recruitment will be approximately 30 participants. However, final numbers will be determined by data saturation, defined as the point at which no new concepts or outcome domains are being identified. To achieve adequate participant enrolment at each site, additional recruiting clinicians will be contacted if needed. Written informed consent will be obtained from all participants.

#### Data collection

The interviews will be conducted face-to-face as first preference or by Skype or Facetime or telephone interviews if preferred by the participant. Each interview will take approximately 40 min and will be audio-recorded and transcribed verbatim. A preliminary interview guide is provided (Appendix 2).

#### Data analysis

Transcripts will be available for participants to review and revise. A summary of the interview findings will be sent to participants for member checking. The transcripts will be imported into software HyperRESEARCH (ResearchWare Inc, http://www.researchware.com, version 3.7.5) for the qualitative data analysis. Two experienced qualitative investigators will supervise the coding and development of descriptive and analytical themes. Using inductive thematic analysis, the findings from the study will be grounded in the participant data [34]. The transcripts will be coded line by line to identify concepts. Similar concepts will be grouped into themes that reflect different outcome domains with the reasons for identifying them. The analysis will be iterative, repetitively moving between the transcripts, analysis and subsequent interviews. The preliminary results will be reviewed and modified by the OMERACT-Adherence steering committee. Conceptual links amongst themes and subthemes will be identified to develop an analytical thematic schema.

### Phase 3: focus groups with modified nominal group technique with patients and caregivers

Patients and their caregivers will be asked to identify outcome domains they regard as important and relevant to measure in trials to support medication adherence, and to discuss the reasons for their choices. A modified nominal group technique will be used to generate a prioritised set of ideas in a group systematically and to encourage the participation of each member [35, 36]. The outcome domains from phases 1 and 2 will be incorporated for discussion and ranking in nominal groups. Additional outcome domains will also be identified in this phase. This study uses both quantitative and qualitative data and has been used successfully in the development of other OMERACT core domain sets [37, 38].

#### Participants and recruitment

At least 12 focus groups (with a minimum of five participants per group) will be convened. Adults aged 18 years and over with gout, osteoporosis or RA and their caregivers will be invited to participate. The recruitment sites and purposive sampling technique are outlined in phase 2. In addition, focus groups will take place in the Netherlands (through Sint Maartenskliniek). Participants who participate in focus groups will be different to those in individual interviews. The groups will be convened until data saturation. The focus groups will be convened by condition at each site. To achieve adequate participant enrolment at each site, additional recruiting clinicians will be contacted if needed. Written informed consent will be obtained from all participants.

#### Data collection

The focus groups will be up to 2 h in duration. An experienced facilitator with training in the nominal group technique and who is not involved in any patient’s care will moderate the groups to encourage open discussion. The questions will be described in an interview guide and discussed among the steering committee [31]. All focus groups will be audio-taped and transcribed verbatim. De-identified transcripts will be available for participants to review and revise. A note-taker will record notes on the interaction among the participants. The preliminary content for the focus group run sheet is provided (Appendix 3).

#### Data analysis

##### Quantitative analysis

An importance score will be calculated for each outcome domain, based on the rankings attributed in the focus groups, to give an overall ranking of all outcome domains identified. The distribution of the ranking for each outcome domain is calculated from the probability of each rank for each outcome domain. The probability has two components: (1) the importance given to the outcome domain by the ranking and (2) the consistency of being nominated by the participants. Higher scores identify outcome domains that are more valued by the participants. These probabilities will be used to compute the weighted sum of the inverted ranking (1/*i*) to obtain the importance score (IS):

$$ \mathrm{IS}=\sum \limits_{i=1}^{\mathrm{no}\ \mathrm{of}\ \mathrm{outcomes}}\mathrm{P}\left({O}_j\ \mathrm{in} \operatorname {rank}\ j\right)\times \frac{1}{i}. $$

The importance scores will also be calculated separately for each condition, as well as for patients and caregivers and compared using a *t*-test with a statistical significance level of *p* < 0.05. Participants who have not ranked at least ten outcome domains will be excluded from this analysis. The analysis will be conducted using statistical software Stata/SE (StataCorp. College Station, TX) and R (R Foundation for Statistical Computing, Vienna, Austria).

##### Qualitative analysis

Transcripts will be imported into HyperRESEARCH (ResearchWare Inc, http://www.researchware.com, software for qualitative data analysis. Using a thematic analysis, the transcripts will be coded line by line by an investigator experienced in qualitative research to identify concepts. Similar concepts will be grouped into themes that reflect the reasons for identifying and ranking the outcome domains. These themes will be discussed by the OMERACT-Adherence steering committee.

### Phase 4: modified Delphi consensus survey

An international online OMERACT-Adherence survey will incorporate all domains identified in phases 1–3 and generate a consensus on up to seven core domains, as well as other domains that may fit under the optional or research domains. Delphi surveys have been used to gain consensus on core domain sets in a range of health conditions [39–42]. The online survey will involve three rounds completed by participants with knowledge, experience or expertise on the topic.

#### Participants and recruitment

Although Delphi surveys used to develop core domain sets for trials in OMERACT have involved up to 250 participants [41–43], there is no agreement on the sample size required for a Delphi survey [44, 45]. To achieve a minimum sample size of 200 respondents at the end of the Delphi survey, by assuming 20% attrition for each round, the initial target sample size will be 390. Participant retention in Delphi rounds will be encouraged with at least two reminder emails. This will include patients and caregivers (minimum *n* = 200); rheumatologists (minimum *n* = 63); pharmacists, nurses, allied health professionals and general practitioners (minimum *n* = 63); outcomes researchers, adherence researchers, clinical trialists, representatives from the pharmaceutical industry and policymakers (minimum *n* = 63).

To achieve adequate participant enrolment, participants will be identified from the networks of the OMERACT-Adherence Group. Following this, a snowball sampling technique will be utilised for recruitment, whereby key informants will be identified for recruitment by existing participants to ensure that a broad range of participant characteristics (including countries and health-care systems) and experiences are captured.

#### Data collection

##### Generating the list of outcome domains

The modified Delphi survey will include outcome domains identified in phases 1 to 3. The survey will include a plain language definition of each listed outcome domain. The survey will be reviewed by the OMERACT-Adherence Group, and piloted with at least three patients, three clinicians and three other relevant stakeholders.

##### Survey administration

The surveys will be completed online using the survey platform Qualtrics (Qualtrics Provo, UT). Each participant will be given a unique identifier so that their responses from each round of the survey can be linked anonymously. A minimum of three reminders will be sent to participants during the Delphi rounds, with the aim of achieving a response rate of at least 70% across all three rounds of those who have agreed to participate.

##### Delphi round 1

Participants will rate each outcome domain using a nine-point Likert scale. Ratings 1 to 3 are not important, 4 to 6 important but not a priority, and 7 to 9 very important and a priority. Unsure will also be an option. Responses will be mandatory and participants will be encouraged to use the full range of scores. The sequence of outcome domains will be randomised to minimise ordering bias. Participants can provide comments for each outcome domain in a free-text box and suggest new outcome domains. All new outcome domains that are suggested will be reviewed by the steering committee and discussed for inclusion in round 2.

Any outcome domain where ≥70% of either patients and caregivers or other stakeholders have rated the outcome domain as very important and a research priority (scores 7–9) will be retained in round 2 and reported back to participants. All items where ≥70% of the participants voted the item as not important (1–3) will be excluded from the Delphi list. All the remaining items and new items will be sent back for re-scoring in round 2.

##### Delphi round 2

Participants will be presented with a graph showing the distribution of scores for all retained domains for (1) patients and caregivers, (2) other stakeholders and (3) all participants. Comments from round 1 by all other participants will also be provided. The participant’s own response from round 1 will be highlighted. Participants will use the same Likert scale for re-scoring. Participants can provide comments for each outcome domain in a free-text box.

Any outcome domain where ≥70% of either patients and caregivers or other stakeholders have rated the outcome domain as very important and a research priority (scores 7–9) will be retained in round 3 and reported back to participants. All items where ≥70% of the participants voted the item as not important (1–3) will be excluded from the Delphi list. All the remaining items will be sent back for re-scoring in round 3.

##### Delphi round 3

Participants will view the distribution of scores and comments for each domain from round 2. Participants will see their own scores from round 2 highlighted and re-score outcome domains. After the rating questions, participants will be asked to complete a Best–worst scale survey [46]. In the best-worse survey, the group will be presented with up to six lists that will contain a subset of six of the outcome domains remaining in round 3. Participants will be asked to choose the most important and least important outcome domains from each list. The Best–worst scaling survey will quantify the relative importance of each of the round 2 outcome domains.

#### Data analysis

The mean, median and proportion of the ratings for each outcome domain from all three rounds will be calculated. The scores will be calculated separately for patients/caregivers and other stakeholders. A Wilcoxon sign rank test or *t*-test will be used to compare the mean difference in rating scores between both stakeholder groups, with a significance value of *p* < 0.05. The Best–worst scale survey will be used to calculate the relative importance score for each of the round 2 outcome domains. Multinomial logistic regression models will be used to calculate a relative importance score for each outcome domain normalised to the range 0 (least important) to 10 (most important). Importance scores will be calculated separately for patients, caregivers and other stakeholders. The influence of demographic factors, such as age, gender and condition, will be investigated. Participants who have not completed all three Delphi rounds will be excluded from the analysis.

Based on previous Delphi surveys used in outcomes research, a preliminary core domain set will be based on the outcome domains for which ≥70% of both patients/caregivers and other stakeholders have rated it as critically important (rating 7–9) [43]. For feasibility, up to seven critically important outcome domains (based on the means, medians and proportions of ratings and importance score) will be identified as the preliminary core domain set.

### Phase 5: consensus workshop

A consensus workshop will review the results from phases 1 to 4 and discuss the potential core domain set. Strategies to develop outcome measures will also be discussed. The target will be at least 60 participants, with a minimum of 20 patients and caregivers. To achieve adequate participant enrolment, the stakeholder workshop is anticipated to occur during the 2020 OMERACT meeting. Invitations will be extended to health professionals (rheumatologists, pharmacists, nurses and other allied health professionals), researchers, policymakers and pharmaceutical industry representatives with expertise in medication adherence in rheumatology. To facilitate implementation, invitees will include health professionals who have key roles in specialty professional organisations, guidelines, registries, journals, regulatory agencies and funding organisations. All parts of the workshop will be audio-recorded and transcribed.

Participants will be sent a copy of the results from phases 1 to 4 prior to the workshop and asked to consider the results to date, so that they are prepared to give informed and considered feedback. The preliminary agenda for the consensus workshop is presented below.

#### Part 1: introduction

The aims, method and the results from OMERACT-Adherence phases 1 to 4, including the preliminary core domain set, proposed consensus definition and strategies to develop outcome measurements, will be presented by the chair of the OMERACT-Adherence Group.

#### Part 2: breakout groups

Participants will be assigned to breakout groups with approximately 12 participants per group (each with a facilitator and co-facilitator chosen from the OMERACT-Adherence Group). The groups will contain a mixture of stakeholders, including a minimum of two patients or caregivers, to promote the exchange of different perspectives. A briefing session, including a detailed run sheet with the question guide, will be provided to train facilitators. The facilitators will moderate the group discussion and take notes to report back to the larger group, focusing on the candidate core domains and strategies to develop outcome measures.

#### Part 3: plenary discussion

The group will reconvene after the breakout group session. Each group will report back the results of their discussion to the wider group. Participants will be encouraged to provide feedback on the issues raised by other groups. The workshop chair will moderate the forum and summarise key points.

##### Finalisation of the core domain set

Final consensus voting will include voting on each proposed domain. Changes to domains (e.g. wording or definition) will be permitted during phase 5. All domains voted for by ≥70% of participants will be included in the core domain set. In addition, attendees will vote on whether appropriate steps outlined in phases 1–4 were followed to obtain the core domain set and agreement on a proposed research agenda for core outcome measurement development. Following the workshop, all transcripts will be entered into the software HyperRESEARCH (ResearchWare Inc. http://www.researchware.com, version 3.7.5). The data will be coded and analysed to identify participant perspectives on the potential core domain set, and suggestions and challenges for implementation. The key findings will be reviewed by the OMERACT-Adherence steering committee prior to submitting a finalised workshop report. Phases 1 to 5 of the OMERACT-Adherence process, including the workshop report on the core domain set, will be published in peer-reviewed journals.

## Discussion

OMERACT-Adherence will use a validated and systematic approach to develop a consensus-based core domain set that OMERACT will recommend is reported in all clinical trials of interventions aimed to improve medication adherence in paediatric and adult rheumatic conditions. The OMERACT-Adherence core domain set may be considered for other contexts including other specialties, and other types of studies such as observational studies in which medication adherence is a key requirement to ensure the optimal uptake of new medications. Once the OMERACT-Adherence core domain set has been ratified by OMERACT attendees, core outcome measurements for each of the core domains will be identified or developed as needed using the OMERACT filter to ensure that measures are truthful, discriminative and feasible [47]. Guidelines for selecting outcome measurements for core domains that have been developed by the Core Outcome Measures in Effectiveness Trials (COMET) and Consensus-based Standards for the Selection of Health Measurement Instruments (COSMIN) initiatives will also be used to guide this process [23, 48].

In addition to publications and research presentations, to facilitate the dissemination and uptake of the OMERACT-Adherence core domains set into clinical trials, national and international stakeholders will be consulted throughout the study phases and at an implementation workshop at the completion of the study. Ultimately, the standardised use of a consensus-based set of high-priority outcome domains will enable all stakeholders to make decisions about strategies to improve medication adherence.

### Study status

Data collection and recruitment commenced for phases 1, 2 and 3 in October 2017. A time schedule has been adapted from the SPIRIT figure and is provided (Appendix 1). The OMERACT-Adherence five-phase study was registered on the COMET database on 27 November 2017 (http://www.comet-initiative.org/studies/details/1068). Any important amendments to the protocol will be discussed amongst the OMERACT-Adherence steering committee and submitted to the COMET database. The date of submission for this protocol (version 1) is 29 November 2017.

### Additional file

Additional file 1: SPIRIT 2013 Checklist: Recommended items to address in a clinical trial protocol and related documents. (DOC 121 kb)

## Figures and Tables

**Table 2:**
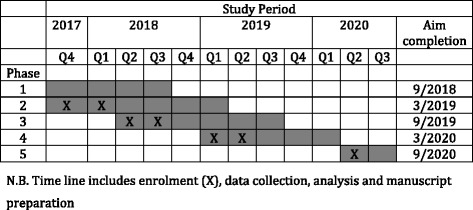
Schedule of study phases

## Appendix 1

**Table 1:** Definitions and examples of intervention functions and sources of behaviour

Intervention functions	Definition	Examples
Education	Increasing understanding or knowledge	Group patient education meetings
Persuasion	Using communication to stimulate action or induce positive or negative feelings	Using motivational interviewing to encourage medication adherence
Incentivisation	Creating an expectation of reward	Payment to complete computer-based interactive adherence programme
Coercion	Creating an expectation of punishment or cost	Punishment system for a child who does not take their medications
Training	Teaching skills	Self-management training
Restriction	Using rules to decrease the opportunity to engage in the target behaviour	Restricting biologic prescriptions to those with adequate adherence
Environmental restructuring	Changing the social or physical context	On-screen prompts to remind rheumatologist to address medication adherence with patients
Modelling	Providing an example for people to imitate or aspire to	Peer educators motivating other patients
Enablement	Increasing means or reducing barriers to increase capability or opportunity (excluding education and training or environmental restructuring)	Alarm device to remind patients to take medications Controlled-release medications to reduce number or frequency of medications
Sources of behaviour		
Capability	The individual’s psychological or physical capacity to engage in the behaviour	Psychological capability (e.g. medication knowledge) Physical capability (e.g. medication-taking skill)
Opportunity	Factors that lie outside the individual that prompt a behaviour or make it possible	Physical opportunity (e.g. cost of medication) Social opportunity (e.g. societal acceptance of medication taking)
Motivation	All the brain processes that energise and direct behaviour	Reflective motivation (e.g. analytical decision-making) Automatic motivation (e.g. immediate emotional response to medication taking)

## Appendix 2

### Preliminary interview guide for interviews with patients and caregivers

1. *Welcome and introduction:* Aspects of treatment that are important and challenging.
2. *Current outcomes:* Presentation and discussion of outcome domains currently reported in interventions to support patients with medication adherence (as identified in phase 1).
3. *Perspectives on outcomes:* Outcome domains the participants believe are important and relevant for trials and the reasons why.
4. *Outcome implementation:* Ideas on how a core domain set can be disseminated to the broader group of stakeholders

## Appendix 3

### Preliminary run sheet for focus groups with patients and caregivers

1. *Welcome and introduction (10 min):* The facilitator will explain the aims of the study, define what outcome domains are in the context of clinical trials, describe the scope of interventions to support medication adherence (Fig. 1) and ask participants to introduce themselves (including their diagnosis and medications).
2. *Focus group discussion (40 min):* Participants will be asked to discuss their experiences and motivation of taking medications for their rheumatic condition, including perceived benefits, harms and complications related to their treatment.
3. *Nominal group technique (70 min):* Each participant will be asked to suggest one to two domains they consider are most important to include in trials of interventions to support medication adherence. The facilitator will ask participants in turn to read out their suggestions, which will be recorded for all participants to see on a whiteboard or flip chart. Once the group has created their list of outcome domains, the facilitator will add outcome domains identified from the systematic review, patient and caregiver interviews, and previous nominal groups. The list of outcome domains will be discussed to ensure that all members understand the meaning of the outcome domains. Participants will then individually rank all the outcome domains in the order of perceived importance, from 1 (most important) to X (least important). If participants have difficulty ranking all the outcome domains, they will be asked to rank their top ten domains. The facilitator will ask participants to read out their top three choices and will note this for all to see and discuss.

Similarities and differences in ranking will be discussed among the participating groups. The outcome domains from each group will be reviewed and discussed among the OMERACT-Adherence steering committee.

## Abbreviations

ACR: American College of Rheumatology
COMET: Core Outcome Measures Effectiveness Trials
COSMIN: Consensus-based Standards for the Selection of Health Measurement Instruments
OMERACT: Outcome Measures in Rheumatology
RA: Rheumatoid arthritis
RCT: Randomised Controlled Trial
